# Mechanism and application of immune interventions in intracerebral haemorrhage

**DOI:** 10.1017/erm.2024.22

**Published:** 2024-10-08

**Authors:** Xiaoxiao Xu, Yuanwei Li, Shiling Chen, Xuan Wu, Jiarui Li, Gaigai Li, Zhouping Tang

**Affiliations:** Department of Neurology, Tongji Hospital, Tongji Medical College, Huazhong University of Science and Technology, Wuhan, China

**Keywords:** immune interventions, immune–inflammatory response, intracerebral haemorrhage, post-stroke immunodeficiency, stroke

## Abstract

Despite stroke being one of the major and increasing burdens to global health, therapeutic interventions in intracerebral haemorrhage (ICH) continue to be a challenge. Existing treatment methods, such as surgery and conservative treatment have shown limited efficacy in improving the prognosis of ICH. However, more and more studies show that exploring the specific process of immune response after ICH and taking corresponding immunotherapy may have a definite significance to improve the prognosis of cerebral haemorrhage. Therefore, immune interventions are currently under consideration as therapeutic interventions in the ICH. In this review, we aim to clarify unique immunological features of stroke, and consider the evidence for immune interventions. In acute ICH, activation of glial cells and cell death products trigger an inflammatory cascade that damages vessels and the parenchyma within minutes to hours of the haemorrhage. Immune interventions that ameliorate brain inflammation, vascular permeability and tissue oedema should be administered promptly to reduce acute immune destruction and avoid subsequent immunosuppression. A deeper understanding of the immune mechanisms involved in ICH is likely to lead to successful immune interventions.

## Introduction

Stroke remains the second-leading cause of death and the third-leading cause of death and disability combined (as expressed by disability-adjusted life-years lost) in the world. Moreover, according to the data from Global Burden of Disease 2019, there were 12.2 million incident cases of stroke in 2019 and intracerebral haemorrhage (ICH) is responsible for 27.9% of all strokes and the highest fatality for all subtypes of strokes (Refs 1, 2). The mortality of patients with ICH is 30–50% within the first year (in-hospital and out-hospital are 32.4 and 45.4%, respectively), and 49.5% among survivors after the first year (Refs 1, 3). Despite stroke being one of the major and increasing burdens to global health, therapeutic interventions in ICH continue to be a challenge and no effective therapy has been identified beyond the overall critical management of ICH (Ref. 4). Furthermore, more than 250 clinical trials, which included more than 1000 brain protector molecules, have failed, demonstrating a critical need for new approaches to developing therapies for acute stroke (Refs 5, 6).

Inflammation and immune responses have emerged as essential elements of the pathobiology of stroke. It is well known that the location of cerebral haemorrhage has a key impact on prognosis; the common locations of cerebral haemorrhage include basal ganglia area, brainstem area, cerebellar area, ventricular area, etc. While the immune system participates in the brain damage caused by ICH, the damaged brain also exerts a powerful immunosuppressive effect which promotes fatal intercurrent infections and threatens the survival of stroke patients (Refs 7, 8). Thus, modulation of adaptive immunity exerts a remarkable protective effect on ICH and offers the prospect of new stroke therapies.

### Immune–inflammatory response after ICH

ICH can cause primary and secondary brain injury. The immediate effects of ICH, such as haematoma mass effect and the consequent increase in intracranial pressure, lead to primary injury, whereas subsequent effects, such as inflammation and brain oedema, contribute to secondary injury (Ref. 9).

Primary brain damage induced by haematoma mass effect develops within the first few hours after ICH. With the expansion of haematoma, the increased intracranial pressure can lead to herniation and death (Ref. 10). Secondary brain damage induced by haematoma toxic effect results from activation of glial cells, leucocytes and other inflammatory cells, which drive proinflammatory, oxidative and cytotoxic cascades causing cell death and functional impairment (Ref. 10). Available evidence from preclinical and clinical studies suggests that the inflammation reaction after ICH is characterized by the accumulation and activation of inflammatory cells and mediators. ICH allows the immediate infiltration of blood components, including red blood cells, leucocytes, macrophages, plasma proteins into the injury site (Ref. 11). The following inflammatory response involves inflammatory mediator release, protease activation, microglia and astrocyte activation, and brain tissue breakdown (Refs 9, 12). Inflammatory cells include blood-derived leucocytes and macrophages, resident microglia, astrocytes and mast cells. Microglia are believed to be the first non-neuronal cells to react to brain injury, they act as guardians of neuronal survival and function under various pathologic conditions in the brain (Refs 9, 10, 13).

Increasing evidence suggests that leucocytes/macrophages, activated microglia and astrocytes are major cellular mediators of secondary brain damage based on their local release of immune-active molecules (cytokines, chemokines, prostaglandins, proteases, ferrous iron and so on) (Refs 14, 15).

#### Microglia

As key innate immune cells, microglia act as guardians of the brain and are recognized to be the first non-neuronal cells to respond to various acute brain injuries (Ref. 16), including ICH (Ref. 9). According to Bai *et al*. (2020), there is mounting evidence that the brain's primary supply of immunomodulatory chemicals such as ferrous iron, prostaglandins, proteases, chemokines and cytokines comes from activated microglia (Ref. 17). These molecules combine to produce secondary brain lesions and subsequent brain repair processes. Classically activated microglia/M1 cells have been strongly associated with worsening cerebral oedema surrounding ICH seen on radiography (Ref. 18). Upon activation, M1 cells transiently develop a primarily phagocytic function to clear necrotic neurons and cellular debris to diminish the deleterious release of inflammatory cytokines and chemoattractants (Ref. 19). However, as the number of M1 cells increases, the phagocytic ability appears to significantly decrease, and there is increased secretion of inflammatory cytokines, chemokines and other neurotoxic mediators leading to widespread cellular damage (Ref. 20). Additionally, oxidative stress from activated microglia likely plays an important role in the impairment of the blood–brain barrier (BBB). While in the face of anti-inflammatory cytokines, specifically interleukin (IL)-4, IL-10 and IL-13, microglia can undergo alternative activation to the M2 phenotype, resulting in haematoma/debris removal, healing (extracellular matrix deposition), neurogenesis/angiogenesis and neuroprotection, which correlate with the resolution of cerebral oedema and neurologic improvement. The manifestation of microglia activation includes their elevation of cytokines and phagocytic receptors, and their change from a ramified to an amoeboid morphology. The activated microglia, over time, become indistinguishable in morphology and by most markers from the monocyte-derived macrophages that infiltrate the central nervous system (CNS), so both are commonly referred to as microglia/macrophages (Ref. 21). In fact, cell therapy with bone marrow-derived mononuclear cells or macrophages ameliorates injury and improves outcome after stroke (Refs 22–24). Given these findings, research on microglial activation and function, as well as cell–cell interactions, is crucial to therapies of ICH.

#### Reactive astrocytes

Astrocytes are vital for normal brain functions and considered to be active elements of the brain circuitry. Calcium signalling in activated astrocytes has been proposed to trigger the release of gliotransmitters, such as glutamate, adenosine triphosphate, tumour necrosis factor (TNF)-*α* and D-serine, which can modulate neuronal excitability, synaptic activity and plasticity (Ref. 25). A new research using a human induced pluripotent stem cell-derived BBB co-culture model shows that TNF transitions astrocytes to an inflammatory reactive state that causes BBB dysfunction through activation of STAT3 and increased expression of SERPINA3, which encodes alpha 1-antichymotrypsin (Ref. 26). Astrocytes react to many CNS injuries and undergo profound morphologic and functional remodelling that is dependent on the type and timing of injury and the distance from the injury site. Disturbance of astrocytic function by brain injury or disease can compromise neuronal functionality and viability. Reactive astrocytes are hallmarks of various neuropathologic conditions (Refs 27, 28). There are several mechanisms by which reactive A1 astrocytes may be detrimental in the setting of ICH. The presence of haemoglobin in the brain parenchyma is a powerful trigger of oxidative stress, which then induces matrix metalloproteinase-9 (MMP-9) on astrocytes (Ref. 29). MMPs have been implicated in blood–barrier injury and subsequent cerebral oedema in ICH (Ref. 30). There are reports showing that MMP-9 was mainly derived from reactive astrocytes after ICH and the massively increased MMP-9 plays a deleterious role in BBB. Moreover, the study identified NDRG2-PPM1A signalling in reactive astrocytes as a key switch for MMP-9 production (Ref. 31). The post-ischaemic induction of MMP-12 in the brain degrades tight junction proteins, increases MMP-9 and TNF*α* expression and contributes to the BBB disruption (Ref. 32). Another consequence of blood extravasation on astrocytes is the presence of thrombin in the extracellular matrix, which also induces cerebral oedema after ICH (Ref. 33) and leads to the activation of PAR-1, which can be primarily found at perisynaptic endfeet of the astrocyte (Ref. 34). In addition to causing further cerebral injury, PAR-1 on astrocytes causes rapid remodelling of synapses, resulting in migration of astrocytic processes away from excitatory glutamatergic synapses (Ref. 35). This reduction of glutamatergic receptors in the setting of astrocytic PAR-1 impairs long-term plasticity (Ref. 35). This detrimental mechanism may be involved in the cognitive impairment seen after ICH. Although there is evidence that suppression of astrocytic activity decreases haematoma volume, BBB destruction and improves neurologic outcomes, therapeutic targets as it relates to astrocytes have not been explored and warrant further investigation (Ref. 36).

#### T lymphocytes

T lymphocyte functions have been well-characterized in ischaemic stroke models (Ref. 37), but their role in ICH is less defined. As such, much of our understanding of T-cell function in ICH is extrapolated from pre-clinical ischaemic models. It is theorized that activated T cells can cross the BBB once a T-cell response is activated in the presence of a CNS autoantigen in the lymphoid organs (Ref. 38). In the ischaemic stroke model, the influx of CD4^+^ and CD8^+^ T cells are thought to play a detrimental role in perpetuating ischaemic tissue injury by contributing to the microvascular dysfunction caused by cerebral ischaemia/reperfusion injury. Ninety-three models of either CD4^+^ or CD8^+^ deficiency resulted in decreased stroke volume and improved neurologic performance (Refs 19, 39). Alternatively, some types of T cells play a neuroprotective role, such as regulatory T cells following ischaemia, which promote recovery via their immunomodulating properties. Another set of functionally opposing lymphocytes involved in the inflammatory response in ICH is Th17 and Treg cells. This set of cells has a reciprocal relationship in the promotion and suppression of inflammation. Th17 cells differentiate in the presence of IL-6, IL-21, IL-23 and TGF-*β*. These cells express CCR6 and ROR*γ*t markers and secrete IL-17, IL-21 and IL-22. It is theorized that a key pathophysiologic mechanism is the disruption of the BBB via binding of cytokines secreted by Th17 cells to IL-17 and IL-22 receptors found on endothelial cells, allowing for the further recruitment and migration of Th17 cells and other pathogenic cell types, including Th1 cells. However, the actual role of Th17 cells in the pathophysiology of post-ICH neuroinflammation remains unknown.

#### Leucocyte

Besides, ICH initiates an inflammatory response characterized by leucocyte recruitment and elevated cytokine levels (Ref. 40). Specific leucocyte populations, including neutrophils, T cells and inflammatory monocytes, promote secondary injury in models of ICH (Refs 41–43). In blood samples from 90 patients with ICH, a lower neutrophil number was associated with a longer survival. Neutrophils are major sources of MMP-9, ROS and TNF-*α*, all of which can be injurious (Ref. 44). In patients with ICH, T lymphocytes are found in the perihaematomal brain but in low concentrations and they might interact with microglia to enhance the activity of microglia (Refs 45, 46). Thus, it is thought that these cells principally inflict damage through the release of reactive oxygen species, pro-inflammatory cytokines and proteases (Refs 42, 47), but the mechanisms used for migration into the CNS after ICH are unclear.

#### Natural killer cells

Perihaematomal oedema (PHO) occurs within hours after ICH, leading to secondary injury manifested by impaired BBB integrity and destruction of adjacent tissue (Ref. 48). Natural killer (NK) cells are the predominant immune cell subset that outnumbers other infiltrating immune cell types during the early stages of ICH. Unbiased clustering of single-cell transcriptional profiles revealed two major NK cell subsets that respectively possess high cytotoxicity or robust chemokine production features in the brain after ICH, distinguishing them from NK cells of the periphery (Refs 49, 50). NK cells exacerbate BBB disruption and brain oedema after ICH via cytotoxicity towards cerebral endothelial cells and recruitment of neutrophils that augment focal inflammation (Ref. 51). Thus, brain-bound NK cells acquire new features that contribute to PHO formation and neurological deterioration following ICH (Ref. 52).

In conclusion, after cerebral insult, inflammation is initially driven by the M1 microglia, secreting cytokines (e.g. IL-1*β* and TNF-*α*) that are involved in the breakdown of the extracellular matrix, cellular integrity and the BBB (Refs 53–55). Additionally, inflammatory factors recruit and induce differentiation of A1 reactive astrocytes and T helper 1 (Th1) cells, which contribute to the secretion of inflammatory cytokines, augmenting M1 polarization and potentiating inflammation. Within 7 days of ICH ictus, the M1 phenotype coverts to an M2 phenotype, key for haematoma removal, tissue healing and overall resolution of inflammation. The secretion of anti-inflammatory cytokines (e.g. IL-4, IL-10) can drive Th2 cell differentiation. M2 polarization is maintained by the secretion of additional anti-inflammatory cytokines by the Th2 cells, suppressing M1 and Th1 phenotypes (Ref. 56). Moreover, according to a newly published study, high dynamic systemic immune-inflammation index was associated with poor 6-month outcomes in patients with basal ganglia ICH (Ref. 57). All the data indicate that immune-inflammation after ICH is crucial for patients' prognosis and treatment.

### Post-stroke immunodeficiency

Stroke-induced immunodepression (SIDS) is an essential cause of post-stroke infections.

**Figure 1.**
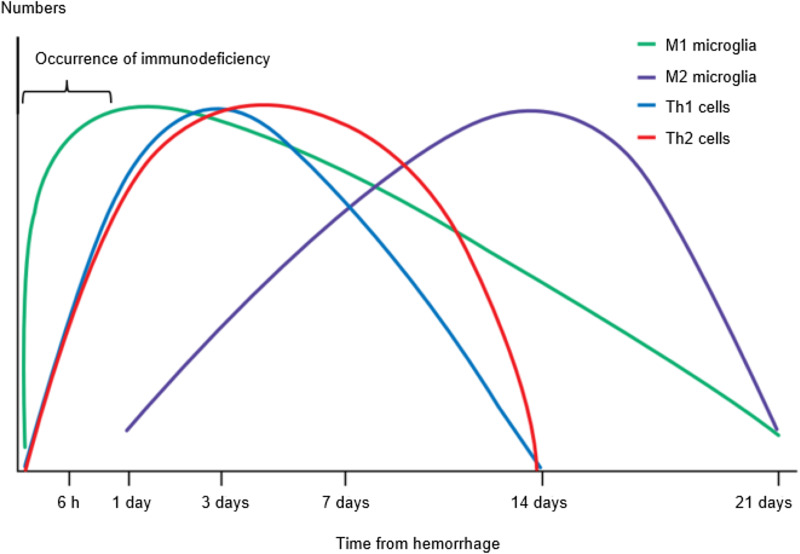
Timeline of immune–inflammatory response after ICH and the occurrence of post-stroke immunodeficiency. Inflammation reaction after ICH is characterized by the accumulation and activation of inflammatory cells and mediators. Inflammatory cells include blood-derived leucocytes and macrophages, resident microglia, astrocytes, T lymphocytes and natural killer cells. Inflammatory mediators include IFN-*γ*, IL-1*β*, IL-6, IL-23, TNF-*α*, IL-12, IL-17 (M1), IL-4, IL-10, TGF-*β* (M2), TNF-*α* (Th1), IL-4, IL-5, IL-13 (Th2) and so on. Immunodepression rapidly occurs within 24 h after stroke onset and persists for weeks, whereas SAP usually occurs between days 1 and 7.

Severe and acute insults to the CNS (such as traumatic brain injuries, spinal cord injuries and strokes) have a significant impact on the immune system. The main clinical manifestations of SIDS are the rapid and persistent depression of cellular immune function, deactivation of monocytes and Th1 cells, Th-mediated lymphopenia (Ref. 58), and increased apoptosis of immune cells in the spleen, thymus and lymph nodes (Refs 59, 60). In the days following acute ischaemic stroke, patients develop lymphopenia and in animals and humans, the size of the spleen is reduced (Refs 61–63) and ICH shares some of these characteristics (Fig. 1).

Inflammatory reactions after stroke promote tissue healing and eliminate necrotic cells, whereas excessive inflammatory reactions may cause secondary damage. Changes in the local and systemic immune system in stroke patients may play an important role in prognosis. This downregulation of immune responses originated in the injured brain avoids autoimmunity against brain antigens that are released during cell death which reduces inflammatory reactions and protects brain tissues. However, immunosuppression varies in its extent according to the volume of the haematoma or oedema and weakens the resistance of the human body against pathogens which leads to high rates of systemic infection in the immediate post-stroke period (Refs 60, 62, 64). Among these complications, pneumonia is the most common type of infection observed after acute stroke, which exhibits the greatest effect on the recovery of neurological function. SIDS is closely related to stroke-associated pneumonia (SAP). Immunodepression rapidly occurs within 24 h after stroke onset and persists for weeks, whereas SAP usually occurs between days 1 and 7 (Ref. 65). As a result of immunodepression, the ability of body to resist pathogens decreases after stroke. The pathogenic bacteria that cause SAP are mostly *Staphylococcus aureus* and various Gram-negative bacilli (Ref. 66), which is one of the main reasons for the aggravation of complications and even the death of stroke patients.

Stroke-induced immunosuppression poses a considerable challenge in the use of immune intervention during the acute phase of stroke, particularly with approaches that are designed to limit immune-mediated brain damage. Immune-based intervention should end within 2 or 3 days of stroke onset, ideally before immunosuppression occurs (Ref. 67). Early-stage intervention could also improve the options for later immune intervention by boosting immunity and promoting neurorepair.

### Possible immune interventions in ICH

#### Perihaematomal oedema (PHO)

What is known to all is that activation of resident and migrant cells fuels the inflammatory process surrounding the haematoma, which causes PHO. The extent of PHO correlates with the activity of inflammatory cytokines and MMP (Refs 15, 68). Inflammation-associated upregulation of specific ion channels leads to ion and water perturbations, PHO and lymphocyte infiltration. PHO exacerbates the mass effect of intracerebral blood and catalyses secondary brain tissue damage and neurological deterioration through secondary ischaemic and inflammatory insults (Refs 68, 69). Attenuation of brain swelling is, therefore, a plausible approach to preventing the destructive effects of PHO and the resultant secondary brain injury. Moreover, several retrospective studies show that the use of statins is able to decrease PHO and improve outcomes of ICH (Refs 70, 71) which could be a feasible immunotherapy for ICH.

Treatment with celecoxib, a selective inhibitor of cyclo-oxygenase 2, after ICH reduced inflammatory cell infiltration, brain oedema and subsequent perihaematomal cell death (Ref. 72). A multicentre trial of celecoxib that included 44 patients also showed that administration of celecoxib in the acute stage of ICH was associated with less expansion of PHO (Ref. 73) but specific mechanism, usage and dosage of celecoxib need further study.

Minocycline seems to be beneficial in a rat model of ICH, even when administered up to 6 h after the insult is induced. Three days after intracerebral blood injection, the extent of brain oedema was lower in rats that received minocycline than in those that received vehicle, and neurological deficits were also reduced (Ref. 74). Minocycline also reduced the number of microglia and macrophages around the haematoma at 5 days after ICH (Ref. 75), preserved capillaries, reduced brain water content and lowered levels of TNF-*α* and MMP-12 (Ref. 76).

peroxisome proliferators-activated receptor-*γ* (PPAR-*γ*) is a ligand-dependent transcription factor that regulates the expression of CD36, which is itself a scavenger receptor that is important for phagocytic activity. In a mouse model of ICH, treatment with PPAR-*γ* agonists, such as rosiglitazone, increased CD36 expression and promoted phagocytosis of red blood cells by microglia and/or phagocytes (Ref. 77). These findings suggest that CD36-mediated phagocytosis is critical in the mechanism by which PPAR-*γ* agonists lead to haematoma resolution (Ref. 78).

Short-term and long-term neurological functions were better in participants who received fingolimod than in participants who did not receive fingolimod. Soon after administration, fingolimod also lowered the number of circulating macrophages, NK cells, CD4 + cells and CD8 + cells, and reduced levels of MMP-9. These effects suggest that fingolimod reduces the migration of these cells and inflammatory mediators to the brain after ICH (Ref. 79). The drug also suppressed the increase in PHO that normally occurs in the first week after ICH and protected the vascular barrier. Collectively, the immune modulation of fingolimod seemed to improve clinical outcomes (Table 1).

**Table 1.** Immune cells in immune–inflammatory response after ICH

Type	Cell	Role
Local immunocytes	Microglia	Main source of immunomodulatory molecules which combine to produce secondary brain lesions and subsequent brain repair processes
	Reactive astrocytes	Calcium signalling in activated astrocytes can trigger the release of gliotransmitters and disturbance of astrocytic function by brain injury or disease can compromise neuronal functionality and viability
Adaptive immunocytes	CD4 + and CD8 + T cells	The influx plays a detrimental role in perpetuating ischemic tissue injury by contributing to the microvascular dysfunction caused by cerebral ischemia/reperfusion injury
	Th17 and Treg cells	This set of cells has a reciprocal relationship in the promotion and suppression of inflammation
Other immunocytes	leucocyte	Promote secondary injury in models of ICH
	NK cells	exacerbate BBB disruption and brain oedema after ICH via cytotoxicity towards cerebral endothelial cells and recruitment of neutrophils that augment focal inflammation

#### Immune–inflammatory response

Much evidence suggests that microglia-mediated inflammation plays a vital role in the ICH-induced brain injury. After ICH, various stimuli could activate microglia and initiate inflammatory response, and subsequently release proinflammatory cytokines and chemokines to increase brain injury. T-regs have been under focus since they could abrogate the pathogenic functions of reactive immune cells and maintain tolerance to self-antigens (Refs 80–82). The first mechanism involves inhibition of cytokine production and/or proliferation of pathogenic T (Teff) cells (Refs 83–85). The second mechanism involves modulation of the cytokine environment at the site of inflammation through direct secretion of cytokines such as TGF-*β* and IL-10 (Refs 86, 87). The third mechanism involves physical elimination of cytotoxic cells, which is considered to have a relatively minor contribution to physiological immune regulation (Refs 88, 89). Moreover, the results of a study which evaluated brain damage and neurological impairments 3 days after ICH demonstrated that Tregs could inhibit brain water content and neurological deficit scores and significantly inhibit expression of TNF-*α*, IL-1*β* and MMP-2 in perihaematoma tissues 3 days after ICH (Ref. 80). All the data suggested that Tregs could inhibit microglia activation in vivo, which means that Treg adoptive therapy is a promising strategy to inhibit inflammation and neurological impairment in ICH and might be utilized in the clinic (Tables 2 and 3).

**Table 2.** SIDS and post-stroke infections

Category	Concrete content
Clinical manifestations	Rapid and persistent depression of cellular immune function, deactivation of monocytes and Th1 cells, Th-mediated lymphopenia, and increased apoptosis of immune cells in the spleen, thymus and lymph nodes
Immune–inflammatory response after ICH	Downregulation of immune responses avoids autoimmunity against brain antigens that are released during cell death which reduces inflammatory reactions and protects brain tissues
Infections	Immunosuppression weakens the resistance of the human body against pathogens which leads to high rates of systemic infection in the immediate post-stroke period

**Table 3.** Possible immune interventions in ICH

Target	Interventions	Mechanism
Perihaematomal oedema	Celecoxib	Reduces inflammatory cell infiltration, brain oedema and subsequent perihaematomal cell death
	Minocycline	Reduces the number of microglia and macrophages around the haematoma, preserves capillaries, reduced brain water content and lowers levels of TNF-*α* and MMP-12
	PPAR-*γ* agonists	Increases CD36 expression and promoted phagocytosis of red blood cells
	Fingolimod	Reduces the migration of these cells and inflammatory mediators to the brain
Immune–inflammatory response	Treg adoptive therapy	Tregs could inhibit microglia activation in vivo
	Block *α*4 integrin function	Blocking *α*4 function may provide neurological benefits by reducing acute inflammation
	Homer1 protein	Homer1 inhibited the phenotypic conversion of astrocytes
Post-stroke immunodeficiency	Aeurosteroid receptor or adrenergic receptor antagonists	Increase the antigen-specific autoreactivity of the central nervous system in experimental stroke patients
	Antibiotics	Protect against infectious complications
	Boost immune function	Use engineered human cytokines to promote recovery of specific lymphocyte populations

While several studies have shown the importance of endothelial cell adhesion molecules, namely vascular adhesion protein-1 and intercellular cell adhesion molecule-1 (ICAM-1), for leucocyte recruitment after ICH (Refs 90, 91), besides, according to a present study *α*4 integrin was elevated on all leucocyte populations in brain after ICH. That means, *α*4 integrin is an important cell adhesion molecule involved in neuroinflammation following ICH and blocking *α*4 function may provide neurological benefits by reducing acute inflammation which is beneficial for the recovery or the prevention of secondary brain damage after ICH (Refs 92–94).

Moreover, according to a newly published study, when Homer1 protein was injected *in situ* into the bleeding site 10 min after ICH, the pathological indices could be effectively improved. Treatment with the Homer1 protein and MAPK inhibitor selumetinib effectively reduced apoptosis in brain tissue at the bleeding site and significantly prolonged the survival time of Homer1^flox/flox^/Nestin-Cre^+/−^ mice within 15 days (test it for up to 15 days). It demonstrates that Homer1 inhibited the phenotypic conversion of astrocytes by inhibiting the MAPK signalling pathway to improve outcome in mice after ICH. However, whether the neuroprotective effect of Homer1 after ICH is due to the short isotype Homer1a remains unclear and needs to be further clarified.

#### Post-stroke immunodeficiency

Therapies aimed at preventing post-stroke immunodeficiency can target three mechanisms. First, neurosteroid receptor or adrenergic receptor antagonists can antagonize lymphocyte apoptosis, reduce infection rate, reduce mortality and improve functional outcomes by increasing the antigen-specific autoreactivity of the CNS in experimental stroke patients (Refs 95, 96).

The second approach is prophylactic administration of antibiotics to protect against infection. This approach is especially relevant for people with a serious stroke who are more susceptible to infectious complications (Ref. 97). A double-blind, randomized controlled trial (The Preventive Antibacterial Therapy in Acute Ischemic Stroke, PANTHERIS) in patients with severe middle cerebral artery stroke suggests that moxifloxacin can reduce post-stroke infections (Ref. 98). A prospective meta-analysis revealed that preventative antibiotic therapy reduced the incidence of infection from 36 to 22% without significantly affecting mortality (Ref. 99), although differences between study populations and designs meant the meta-analysis had insufficient power to draw a definitive conclusion about differences in mortality.

The third, and possibly the most physiological, approach is to boost immune function in patients with stroke, but this approach has not been attempted in experimental models or patients. The principle is to use engineered human cytokines to promote recovery of specific lymphocyte populations that are depressed by stroke. When to initiate this therapy and which types of cells or immune components to target are unclear.

### Relevant clinical trials

#### Celecoxib

A 2013 multicentre trial investigated the effect of celecoxib in patients with ICH. The intervention group (*n* = 20) received celecoxib (400 mg twice a day) for 14 days. The control group (*n* = 24) received the standard medical treatment for ICH. The primary endpoint was the number of patients with a change in the volume of PHO from the 1st to the 7th ± 1 day (cut-off value, 20%). The result showed that in the primary endpoint analysis using cut-off values, there was a significant shift to reduced expansion of PHO in the celecoxib group (*P* = 0.005) and with respect to the secondary endpoints, there was also a significant shift to reduced expansion of ICH in the celecoxib group (*P* = 0.046). In addition, the expansion rate of PHO at follow-up tended to be higher in the control group than in the celecoxib group (90.6 ± 91.7% versus 44.4 ± 64.9%; *P* = 0.058). Their study indicated that administration of celecoxib in the acute stage of ICH was associated with less expansion of PHO (Ref. 73).

Moreover, high-dose celecoxib (400 mg twice daily) for 14 days has been shown to reduce PHO and haematoma enlargement in patients with ICH, but without improvement in long-term functional outcome, which may be confounded by the heterogeneity of haematoma location. A 2022 case report involving two cases which use low-dose celecoxib to treat patients with ICH showed that patients' consciousness was improved after low-dose celecoxib (200 mg once daily) administration for 3 and 2 days in case A and B, respectively. Furthermore, other symptoms that concomitantly improved included poor appetite caused by PHO involving the left hypothalamus in case A, and limb weakness caused by PHO of the internal capsule in case B. These cases revealed that low-dose celecoxib may be an effective management for symptoms caused by PHO in patients with ICH, particularly those involving the thalamus (Ref. 100).

#### Minocycline

Minocycline is an MMP-9 inhibitor that may attenuate secondary mechanisms of injury in ICH but the feasibility and safety of minocycline in ICH patients are remained to be evaluated (Ref. 101). According to a meta-analysis of randomized clinical trials (RCTs) which comprised a total of 426 patients, minocycline demonstrated a favourable trend towards 3-month functional independence (mRS-scores of 0–2) (RR = 1.31; 95% CI 0.98–1.74, *P* = 0.06) and 3-month BI (MD = 6.92; 95% CI −0.92 to 14.75; *P* = 0.08). It showed that minocycline administration was not associated with an increased risk of mortality, recurrent stroke, myocardial infarction and haemorrhagic conversion and seems to be a promising neuroprotective agent in acute stroke patients (Ref. 102).

#### Fingolimod

A 2015 study attempted to investigate the effects of fingolimod on inflammatory mediators and vascular permeability in the clinical trial of oral fingolimod for ICH. The results showed that fingolimod decreased the numbers of circulating CD4(+) T, CD8(+) T, CD19(+) B, NK and NKT cells and they recovered quickly after the drug was stopped. The plasma ICAM level was decreased, and IL-10 was increased by fingolimod. Interestingly, fingolimod protected vascular permeability as indicated by a decreased plasma level of MMP9 and the reduced rT1% (Ref. 79).

In another two-arm proof-of-concept study, they included 23 patients with primary supratentorial ICH with haematomal volume of 5–30 ml and gave all patients standard management alone (control participants) or combined with fingolimod (FTY720, Gilenya), 0.5 mg, orally for 3 consecutive days. The result showed that patients treated with fingolimod exhibited a reduction of neurologic impairment compared with control individuals, regained a Glasgow Coma Scale score of 15 by day 7 (100 versus 50%, *P* = 0.01), and had a National Institutes of Health Stroke Scale score reduction of 7.5 versus 0.5 (*P* < 0.001). Neurologic functions improved in these patients in the first week coincident with a reduction of circulating lymphocyte counts. At 3 months, a greater proportion of patients receiving fingolimod achieved full recovery of neurologic functions, and fewer reported ICH-related lung infections. PHO volume and rPHO were significantly smaller in fingolimod-treated patients than in control individuals (Ref. 103).

#### Tranexamic acid

Tranexamic acid, a widely available antifibrinolytic agent with an excellent safety profile, has been shown to reduce haematoma expansion with no increase in thromboembolic complications. A TICH-2 (tranexamic acid for hyperacute primary ICH) study, which was a pragmatic, phase III, prospective, double-blind, randomized placebo-controlled trial showed that tranexamic acid did not affect a patient's functional status at 90 days after ICH, despite there being significant modest reductions in early death (by 7 days), haematoma expansion and SAEs, which is consistent with an antifibrinolytic effect. Tranexamic acid was safe, with no increase in thromboembolic events (Ref. 38).

Another ongoing phase II randomized placebo-controlled double-blind multicentre trial was started since 2022 to determine the efficacy and safety of intravenous tranexamic acid in patients with ICH within 2 h of onset. Participants would receive 1 g intravenous tranexamic acid over 10 min, followed by 1 g intravenous tranexamic acid over 8 h: or matching placebo. The primary efficacy measure is the proportion of patients with haematoma growth by 24 ± 6 h, defined as either ≥33% relative increase or ≥6 ml absolute increase in haematoma volume between baseline and follow-up CT scan. The ultimate objective of this study is to investigate tranexamic acid as a potential therapeutic to acutely attenuate ICH growth (Ref. 104). This is an inexpensive, safe and readily available drug, which can be easily delivered in a variety of settings including mobile stroke units (MSUs). Such a therapy could potentially have significant and widely generalizable implications for the treatment of devastating condition.

A review of current evidence and ongoing trials screened 268 publications and retrieved 17 articles and found out that two randomized controlled trials (RCTs) comparing intravenous tranexamic acid to placebo (*n* = 54) reported no significant difference in death or dependency. Three observational studies (*n* = 281) suggested less haematoma growth with rapid tranexamic acid infusion (Ref. 39). All the results indicate that tranexamic acid is a promising haemostatic agent for ICH, but we await more results of clinical trials before definite conclusions can be drawn.

### Summary

Evidence suggests that targeting inflammation and immune responses could be a viable approach to rescuing brain tissue and improving outcomes after stroke. However, immune–inflammatory response and post-stroke immunosuppression also pose new challenges to immunotherapy. Up to now, only one clinical trial has assessed the effects of immune modulators in ICH. Most of those trials were early-phase, proof-of-concept studies, but gave promising preliminary results. Moreover, with the progress of science and technology and medical level and additional strategies that target immune–inflammatory response and in the very early phase of haemostasis, we will have a deeper understanding of the immune mechanism related to ICH, so as to provide exciting new promise in the immune interventions of ICH in the foreseeable future.
